# X-ray Induced Electric Currents in Anodized Ta_2_O_5_: Towards a Large-Area Thin-Film Sensor

**DOI:** 10.3390/s24082544

**Published:** 2024-04-16

**Authors:** Davide Brivio, Matt Gagne, Erica Freund, Erno Sajo, Piotr Zygmanski

**Affiliations:** 1 Brigham and Women’s Hospital, Boston, MA 02115, USA; pzygmanski@bwh.harvard.edu; 2 Harvard Medical School, Boston, MA 02115, USA; 3 Department of Physics and Applied Physics, University of Massachusetts Lowell, Lowell, MA 01854, USA; 4 RayWatch Inc., Hopkinton, MA 01748, USA

**Keywords:** radiation sensor, Ta pentoxide, thin-film detectors

## Abstract

Purpose: We investigated the characteristics of radiation-induced current in nano-porous pellet and thin-film anodized tantalum exposed to kVp X-ray beams. We aim at developing a large area (≫cm^2^) thin-film radiation sensor for medical, national security and space applications. Methods: Large area (few cm^2^) micro-thin Ta foils were anodized and coated with a counter electrode made of conductive polymer. In addition, several types of commercial electrolytic porous tantalum capacitors were assembled and prepared for irradiation with kVp X-rays. We measured dark current (leakage) as well as transient radiation-induced currents as a function of external voltage bias. Results: Large transient currents (up to 50 nA) under X-ray irradiation (dose rate of about 3 cGy/s) were measured in Ta_2_O_5_ capacitors. Small nano-porous Ta and large-area flat Ta foil capacitors show similar current–voltage characteristic curve after accounting for different X-ray attenuation in capacitor geometry. The signal is larger for thicker capacitor oxide. A non-negligible signal for null external voltage bias is observed, which is explained by fast electron production in Ta foils. Conclusions: Anodized tantalum is a promising material for use in large-area, self-powered radiation sensors for X-ray detection and for energy harvesting.

## 1. Introduction

Tantalum pentoxide (Ta_2_O_5_) has received considerable attention as a protective coating material for chemical equipment, as anti-reflective coating for optical devices, and as thin oxide for nano-porous sintered capacitors for storage in very large-scale integrated circuits [1,2]. Tantalum capacitors are also used in energy harvesting applications [3] as well as in dynamic random-access memory (DRAM) chips [2]. Solid tantalum capacitors have been widely used in electronics, including military and aerospace applications, for more than 20 years. Due to the excellent dielectric properties, the fabrication of flat porous Ta_2_O_5_ films with a large surface area and high specific charge seems to be very promising for applications in electronic and sensor devices [2]. In addition to other deposition techniques, such as physical vapor deposition (PVD) and chemical vapor deposition (CVD), Ta_2_O_5_ films have been grown by anodization [4,5]. Anodization of tantalum has been widely investigated in sulfuric acid, phosphoric acid, and Na_2_SO_4_ solutions at voltages typically up to 200 V. Under these conditions, if the potential is kept below the dielectric breakdown potential, a uniform layer of amorphous Ta_2_O_5_ is obtained. The growth of the layers is generated by a high-field ionic transport process. The oxide films thicken linearly with the increase in the electrode potential at a rate of about 1.6–2.0 nm/V depending on the electrolyte temperature. The anodizing breakdown voltage of the dielectric film is typically in the range of 200–400 V, and it depends on the composition and concentration of the electrolyte, but it is almost independent of current density, temperature, surface roughness, and hydrodynamic conditions [6].

Porous sintered tantalum electrolytic capacitors are manufactured from a powder of pure tantalum metal [1]. The typical particle size is between 2 μm and 10 μm. The powder is compressed under high pressure around a tantalum wire to form a “pellet”. The wire is the anode connection to the capacitor. The pellet is subsequently sintered at high temperature, 1200–1800 °C. This structure is of high mechanical strength and density, but it is also highly porous, having a large internal surface area. Larger area yields larger capacitance. Appropriate selection of the powder grain size, shape and the sintering temperature can control the surface area. The dielectric is then formed over the entire tantalum surface by the electrochemical process of anodization in a weak solution of phosphoric acid. The dielectric thickness is controlled by the voltage applied during the anodization process (formation voltage V_f_). The oxide forms on the surface of the tantalum but it also grows into the material. For each unit of oxide thickness, two thirds grow outward and one third grows inward. The tantalum pentoxide dielectric grows at a rate of 1.7 nm/V [1]. Electrolytic capacitors are characterized by the capacitance value, C, and by the working voltage, V_w_, which is the maximum voltage that can be safely applied to the capacitor, and it is usually 1/3 (or 1/4) of the formation voltage. The next step is the formation of the cathode. Contact from the dielectric to the cathode plate must then be made through a medium that can conform to the dielectric surface. Sintered Ta electrolytic capacitors are typically produced either with manganese dioxide or with conductive polymer. The “pellet” is then dipped into graphite and silver to provide a good connection to the cathode plate. The use of polymer (that does not include oxygen, contrary to MnO_2_ standard solid tantalum cathode) avoids the risk of thermal ignition. Both MnO_2_ and conductive polymer have shown self-healing properties when local shorts occur.

Tantalum capacitors exhibit various degrees of DC leakage, dependent on the capacitor rating (capacitance-voltage), voltage applied, the charging period, and ambient temperature. Tantalum capacitors are polar devices, meaning that the leakage current–voltage characteristic plot is asymmetric. Such polarity is due to the presence of a semiconducting tantalum oxide region between the pentoxide tantalum and the tantalum metal [1]. This semiconducting layer is what makes anodized Ta an interesting material to be exploited as a radiation sensor.

### 1.1. Leakage Charge Transport

Studies with metal–insulating-metal structures identified Ta_2_O_5_ leakage current as composed of several mechanisms: a polarization current attributed to dielectric relaxation [7], a conduction current attributed to Fowler–Nordheim Tunneling at low temperature and Poole–Frenkel mechanisms at higher temperature [8], and a resistance degradation attributed to ionic diffusion that agrees with Space-Charge Limited theory [9]. Poole–Frenkel was determined to be the dominant conduction mechanism under reverse voltage bias [10]. To clarify the various conduction mechanisms, current–voltage studies have been reported in conjunction with temperature-dependent measurements of activation energy of the leakage current [9]. A linear relationship should be obtained if we plot current density, J, and the applied electric field, E, as ln(J/E) vs. (E^1/2^) (Poole–Frenkel effect), ln(J) vs. (E^1/2^) (Schottky emission) and ln(J/E^2^) vs. (1/E) (Fowler–Nordheim tunneling process) [8,9].

Charge transport in amorphous Ta_2_O_5_ films was investigated for anodized films, and for and PVD or CVD deposition techniques [11]. Oxygen vacancies defects concentration was suggested as the main cause of the transient and steady state leakage current. Oxygen vacancies in the Ta_2_O_5_ are mainly located in a thin layer (~15 nm) close to the Ta metal; their concentration can be increased with annealing or by using a reactive counter electrode material (e.g., Al, Ti, Ta). The IV-curve and the photoconductivity under UV and optical irradiation were used to characterize the defect band properties [11]. Transition from an insulating state to a defect band state occurs when the defects occupy about 30% or 40% of the width of the sample. The photoconductivity at 3 eV (below the 4-eV band gap) indicates that the defect density is in the order of 10^19^–10^20^ cm^−3^. This implies a separation between defects on the order of 20–40 Å if the defects are uniformly distributed, sufficiently close for tunneling to occur between defects.

### 1.2. UV/X-ray Response

Radiation-induced conductivity (RIC, σ_RIC_) in insulators depends on the type of charge carriers created by ionization and their respective modes of transport with recombination, trapping and formation of space charge regions. Typically, RIC is proportional to dose rate γ (σ_RIC_ = k_RIC_ γ^Δ^) and to a material-dependent constant, k_RIC_, Δ [12,13]. It must be noted that Ta_2_O_5_ is a high-atomic number (high-Z) and high-density insulator with fairly small band gap (~4 eV), and it has large photoelectric absorption and Compton scattering cross sections. The larger absorption of radiation in Ta_2_O_5_ have been recently proposed and utilized in multilayer dielectric radiation sensitive field-effect transistors [14].

Radiation hardness is an essential parameter for electronic circuits, which can be employed in hostile environments. As the radiation passes through the insulator, hole–electron pairs are generated. Since tantalum pentoxide could be potentially used in such conditions, the radiation effects on MOS devices with Ta_2_O_5_ as an insulator were investigated by some authors [15,16] but inconsistent changes of the leakage current density (either an increase or decrease) were observed before and after irradiation.

Transient prompt-dose effects in high energy density capacitors are also of interest in harsh radiation environments, such as prompt-gamma and X-ray irradiation in the order of 10 Gy in 20 ns radiation burst, where dielectric breakdown can be catastrophic [17]. Response of metal–insulator-metal (MIM) structures under UV [18] or X-ray [19] for radiation detection have been investigated in the past. Strong X-ray induced bulk photovoltaic effect has also been shown in thick anodized Ta_2_O_5_ cells (0.1–3.5 µm oxides) with Ta and Au electrodes [19]. The effect of radiation in Ta/Ta_2_O_5_ film with electrolytic counter electrodes has shown linear relation between photocurrent and radiation intensity at low dose rate [20]. The response of tantalum capacitors under 35 to 40 MeV electron irradiation has also been explored [21].

### 1.3. Ta_2_O_5_ Capacitors as a Radiation Sensor

While these past studies pointed towards a possible use of tantalum oxide films in radiation detection, a more systematic examination of the radiation and electrical properties is lacking in both photovoltaic and photoconductive regimes. We studied a wide range of capacitance/working voltage combinations for commercial electrolytic capacitors and fabricated our own flat thin-film anodized Ta capacitors. Photovoltaic properties of the flat surface of thin Ta_2_O_5_ capacitors may be employed for self-powered large-area thin-film radiation monitoring devices.

In this work we aim to systematically characterize the X-ray response of solid nano-porous electrolytic capacitors, as well as large-area thin-foil flat tantalum capacitors. Because tantalum is a high-Z element, we address RIC properties in view of the relative photoelectric vs. Compton contributions for thin film structures.

Our focus in this manuscript is the radiation response of Ta_2_O_5_ capacitors in view of the development of large area Ta_2_O_5_ anodization electrodes. In the past, only small samples of Ta_2_O_5_ have been made and studied from a solid-state physics or electronic structure perspective [18,19]. Here, we are paving the way towards developing large area (≫1 cm^2^) anodized Ta_2_O_5_ sheets for radiation detection on large surfaces.

To our knowledge, this is the first study comparing the radiation response of commercial tantalum capacitors in the form of pellets to thin film anodized tantalum, with characterizations aiming at developing a radiation detector rather than describing the radiation hardness of capacitors.

## 2. Material and Methods

### 2.1. Fabrication

We studied two types of tantalum pentoxide capacitors: the standard nano-porous electrolytic Ta capacitors (NP-TaC) and large surface area flat thin-film anodized Ta foils (FTF-TaC). Electrolytic Ta capacitors were purchased (Kyocera–AVX, Fountain Inn, SC, USA) and used as a standard reference for thin film capacitors. Off-the shelf porous electrolytic capacitors (Kyocera AVX series TAJ, TCJ) were fabricated by sintering and anodization of tantalum pellets with different granule sizes (proprietary) and with either MnO_2_ (TAJ series) or conductive polymer (TCJ series) as counter electrode. They possessed disparate capacitances (0.1 μF to 22 μF) and working voltages (V_W_ = 2.5–50 V). Pellet sizes ranged between 0.3 mm^3^ and 3.8 mm^3^. Ta_2_O_5_ thicknesses were estimated to be in the 10–260 nm range. We also fabricated large-area Ta-Ta_2_O_5_ conductor films by anodization of 50 μm-thick Ta foils (2 cm × 5 cm size). For counter electrodes a conductive polymer was deposited (Clevios K WUW4324, Heraeus Holding GmbH, Hanau, Germany). Clevios K is a capacitor grade aqueous PEDOT:PSS formulation (typical 2% solids, 50–100 mPas, pH 2). It is ready to be used (dip-coating, doctor blading) and suitable for outer conductive polymer coatings of tantalum capacitors because it conforms to the surface. Drying and curing at slightly elevated temperatures (80–130 °C) was applied for 15 min. Counter electrodes of about 1 cm^2^ were deposited at different locations on the anodized foil.

### 2.2. Current Voltage Measurements

**Figure 1:**
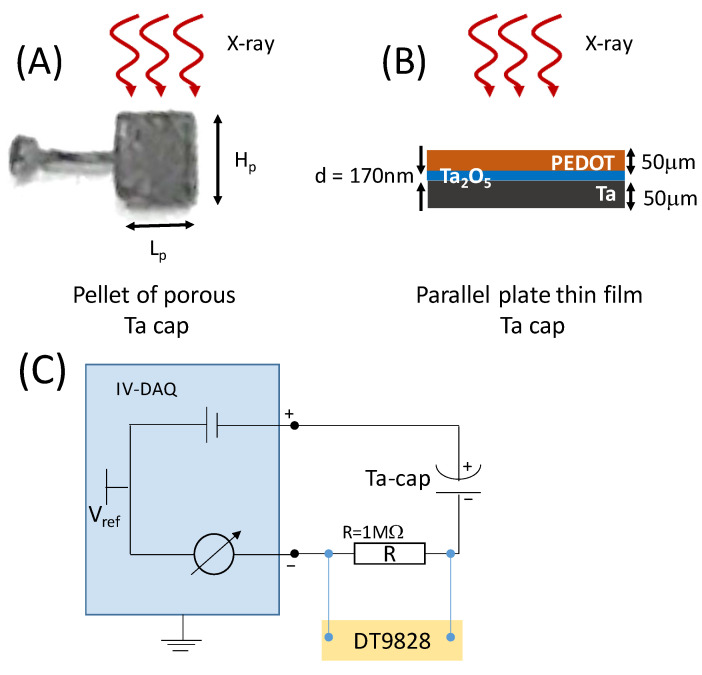
Ta capacitor types: (**A**) picture of a bare pellet of anodized nano-porous tantalum capacitor (NP-TaC); H_p_, L_p_ and W_p_ are the physical dimensions of the pellet. (**B**) large-area flat thin-film Ta capacitor (FTF-TaC) made by anodizing a 50 μm Ta foil; the thickness *d* of the Ta_2_O_5_ was 170 nm; counter electrode is made of PEDOT conductive polymer. (**C**) Schematic of the experimental circuit for current–voltage measurements.

Current–voltage curves were measured using a USB-controlled data acquisition system (IV-DAQ) with modifications in the potentiostat/galvanostat as described in Dobbelaere et al. [22], and by using a DT9828 DAQ (Measurement Computing Corporation, Norton, MA, USA) with 1 MOhm shunt and DC power supply. The IV–DAQ device applies voltages between −35 V and +35 V and measures current in the range between −250 nA and +250 nA with 22-bit resolution, yielding a granularity of 0.12 pA. Schematics of the two capacitor types are shown in Figure 1A,B and a sketch of the experimental circuit is shown in Figure 1C. The noise of the open circuit device is about 1 pA (peak-to-peak) but, as the capacity increases, so does the noise level, because noise gets capacitively coupled from the voltage source to the transimpedance amplifier. The noise therefore increases with the capacitance of the device at about 5 pA/nF. For very large capacitance, the noise saturates to 2.5 nA for 10 µF, and it can be slightly decreased by adding a series resistor at the cost of increasing the RC of the system. This would make the response of the capacitor very slow. We therefore selected capacitance and shunt resistance values that resulted in a good compromise between noise and RC response of the measurement circuit. When these conditions were not possible for the thinnest capacitors, we connected N capacitors of the same type in series (N = 5), maintaining the polarization, so that RC is decreased by a factor of N = 5. In the latter configuration, the current flowing in the circuit is the smallest current between the five capacitors, both for dark current (leakage) and under irradiation. On the other hand, the effective working voltage of the series of N capacitors is N times the working voltage of one of them.

Both, porous NP-TaC and large area flat thin-film capacitors FTF-TaC, were tested by examining their current–voltage curves with and without X-ray radiation. Irradiations were performed using an X-ray system (Ximatron, Varian Medical Systems, Palo Alto, CA, USA) with 120 kVp tube voltage, varying tube current (mA) and varying source-to-surface distance. When operated at 120 kVp and 25 mA, this device produces 3.80 ± 0.02 mGy/s air kerma rate at 100 cm from the source, which was measured using a commercial ionization chamber (DCT10-MM, IBA Dosimetry, Bartlett, TN, USA) that was calibrated by the manufacturer. The calibration was verified with an independently calibrated RadCal 10X6-3CT ion chamber (RadCal, Monrovia, CA, USA).

We acquired a signal under irradiation for several fractions of the rated working voltage, V_W_, of each capacitor between *V*/*V_W_* = −0.05 and *V*/*V_W_* = 0.9. Since *V_W_* is proportional to the thickness of the oxide, the fraction *V*/*V_W_* is proportional to the electric field inside the oxide.

The leakage current as a function of applied voltage was acquired after 120 s initial stabilization period.

### 2.3. Relations between Capacitor Parameters

The oxide thickness, *d*, is proportional to the anodization formation voltage *V_f_*, while the working voltage, *V_W_*, of a capacitor is defined as 1/3 of the formation voltage. The anodization growth rate, *α*, is about 1.7 nm/V [1].

(1) d=α⋅Vf=α⋅3⋅VWα=1.7 nm/VVW=13⋅Vf

The effective area of the capacitor is calculated from the formula:

(2) $$A=\frac{C\cdot d}{\varepsilon_{0}\varepsilon_{r}}$$

where *C* is the measured capacitance and *ε_r_* = 27 (unitless) for Ta_2_O_5_ [1].

The quantity *CV*/*g* is often used by manufacturing companies to characterize the Ta powder grain type, and it is a is a measure of the capacitor volumetric efficiency, which depends on *Ta* particle size and shape [1]. This quantity is proportional to the ratio of the effective surface area/pellet volume.

(3) $$“CV/g”=\frac{C\cdot V_{f}}{m_{Ta}}\propto \frac{A_{eff}}{vol_{p}}$$

where *m_Ta_* is the mass of *Ta* in the pellet and *vol_p_* is the physical volume of the pellet.

## 3. Results

### 3.1. Nano-Porous Electrolytic Tantalum Capacitor (NP-TaC)

**Figure 2:**
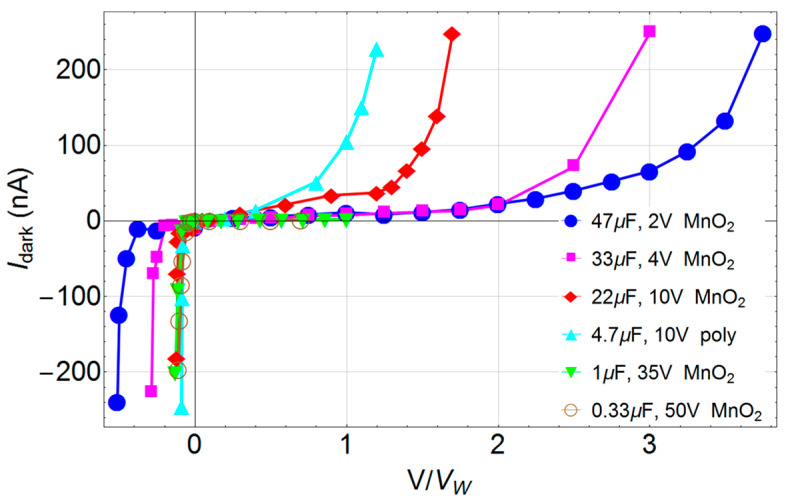
DC dark leakage current IV-curves for different fractions of the working voltage *V_W_* (after waiting time of 120 s) for various types of commercial nano-porous electrolytic tantalum capacitor (NP-TaC). The legend indicates, “capacitance value, *V_W_*, counter electrode material”.

Ta capacitors are polarized devices, therefore the IV-curve at the forward and reverse biases are different in nature, unless double-sided flat geometry is used. The forward leakage current of Ta capacitors follows the power law ~t^−n^, which is known to increase with rated voltage and with capacitance [11,23]. In Figure 2, DC leakage current–voltage curves for several porous electrolyte capacitors (NP-TaC) are reported. Leakage current without radiation (I_dark_) is reported as a function of working voltage fraction.

**Figure 3:**
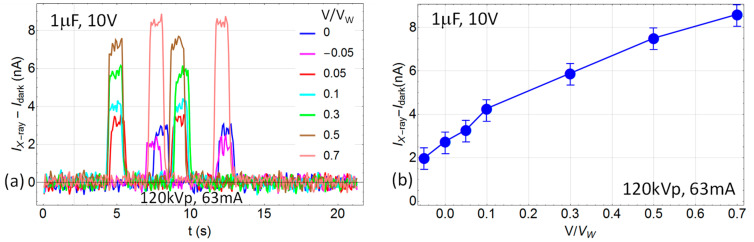
1 μF, 10 V NP-TaC capacitor under irradiation with 120 kVp, 63 mA, 63 mAs at source-to-surface distance (SSD) of 57.5 cm, as a function of time (**a**) and for different fractions of the working voltage (**b**). In (**b**), the signal I(t) is averaged over the two X-ray pulses after subtraction of the dark current (leakage).

In order to keep the dark leakage current low during X-ray irradiation, we kept the voltage below the working voltage (*V*/*V_W_* < 1). In Figure 3a the time response to X-ray radiation of a 1 μF, 10 V NP-TaC is shown for various fractions of applied working voltage. The noise during X-ray irradiation is similar to the dark leakage noise, which is a property of the DAQ circuit for the specific RC of the tested circuit. In Figure 3b, the average current under irradiation is shown as a function of the working voltage fraction *V*/*V_W_* for the same 1 μF, 10 V NP-TaC.

**Figure 4:**
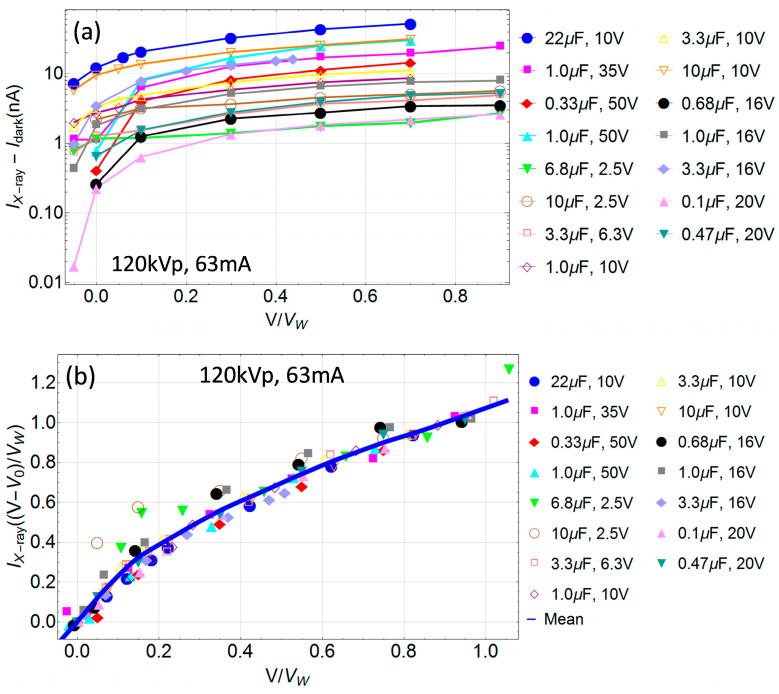
(**a**) Signal as a function of the fraction *V*/*V_W_* under irradiation with 120 kVp, 63 mA, 63 mAs at SSD = 57.5 cm, averaged over the two X-ray pulses after subtraction of the dark current (leakage) for several commercial NP-TaC capacitors. (**b**) Same data as in (**a**) but renormalized to the value at 0.9 *V*/*V_W_* after subtraction of the internal built-in potential (*V* → *V* − *V*_0_).

In Figure 4a the average signal after subtraction of the leakage current as a function of the fraction *V*/*V_W_* is presented for all NP-TaC capacitors. In Figure 4b, the same data are shown but with the shifted voltages after subtraction of the internal built-in potential *V*_0_ (*V* → *V* − *V*_0_) and normalized to the value at 0.9 *V*/*V_W_*. Remarkably, most of the IV-curves are aligned. Two of them have larger signals for small current when compared to the other normalized plots; these correspond to the thinnest oxide layers and large noise (i.e., the smallest *V_W_* of 2.5 V).

### 3.2. Accounting for Varying X-ray Absorption in Nano-Porous Ta Structure

Nano-porous tantalum pellets can be as thick as 0.6–1.4 mm and they absorb up to about 95% of the 120 kVp radiation in the initial 350 μm bulk material, based on NIST mass absorption coefficients [24] for the 120 kVp spectrum obtained using the Spekcalc software V1.1 [25]. Therefore, in NP-TaC capacitors, not all the capacitor volume is equally “active” and most of the volume does not contribute to the radiation-induced current (RIC) but rather absorbs it, hence reducing the efficiency of RIC generation. After disassembling the capacitor cases, we measured the physical thickness (*W_p_*, *L_p_*, *H_p_*) of the tantalum pellets for accounting of the actual active area using the following:

(4) $$A_{Active}=\frac{350\mu m}{H_{p}(\mu m)}\cdot A$$

where *A* is the effective area calculated using Equation (2) and *H_p_* is the thickness of the Ta pellet. Here, the 350 µm value was chosen as the thickness absorbing 95% of the radiation.

We calculated the “CV/g” value of all the capacitors (see Equation (3)) considering the measured capacitance C, the formation voltage *V_f_*, the pellet volume $vol_{p}=W_{p}\cdot L_{p}\cdot H_{p}$ and tantalum density of 16.65 g/cm^3^. This value matters because the attenuation/absorption of X-rays can be very different for different Ta particle sizes and shapes. Therefore, we classified the capacitors based on the “CV/g” value in three groups:

(5) $$\begin{array}{c} “\mathrm{CV}/g”<4000\;\mu F\;V/g;\\ 4000\;\mu F\;V/g<“\mathrm{CV}/g”<9000\;\mu F\;V/g;\\ “\mathrm{CV}/g”>9000\;\mu F\;V/g; \end{array}$$

**Figure 5:**
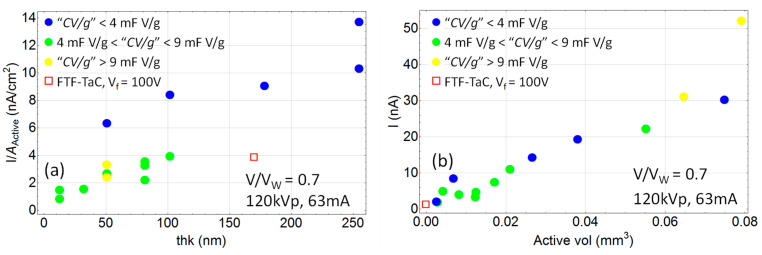
Oxide thickness (**a**) and oxide volume (**b**) dependence of the signal under irradiation with 120 kVp, 63 mA, 63 mAs at SSD = 57.5 cm and with *V*/*V_W_* = 0.7 applied on all capacitors. The active area is calculated using Equation (4), and the active volume by multiplying the oxide thickness by the active area. The red square represents the flat thin-film Ta capacitor (FTF-TaC) made by anodizing a 50 μm Ta foil at a formation voltage of *V_f_* = 100 V (for FTF-TaC, the active area is the physical area).

With this classification, in Figure 5 the thickness and volume dependence of the signal acquired under X-ray irradiation are shown. Uncertainties in these plots arise from: capacitance accuracy (manufacturer provides accuracy within 10%), number of capacitors in the series (N = 1 vs. 5), set-up orientation of the caps with respect to the X-ray beam (10–20% error since they are very small and it is difficult to accurately align them orthogonally to the beam), and larger noise from the DAQ for larger capacitances. Considering all these multiple sources of error, while the data is noisy there is a clear separation of different groups and a quasi-linear relation of current density (nA/cm^2^) and oxide thickness and volume for each group.

### 3.3. Anodized Flat Thin-Film Capacitor Foils (FTF-TaC)

**Figure 6:**
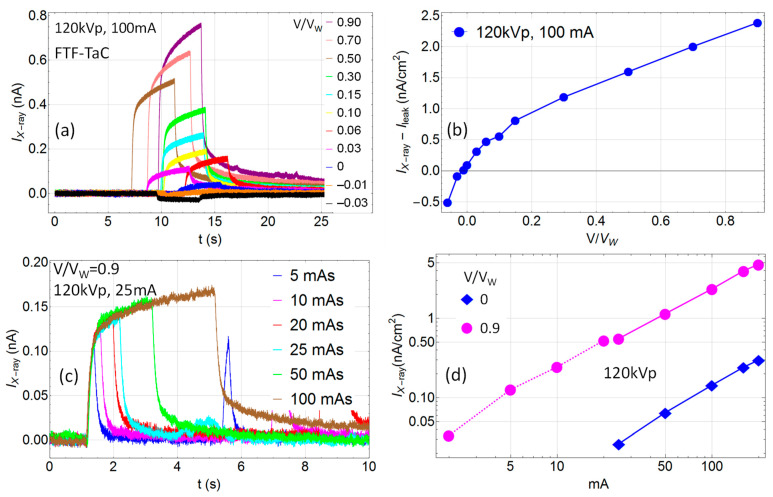
Flat Thin-Film tantalum foil (FTF-TaC) anodized at the formation voltage of *V_f_* = 100 V. The area of the counter electrode was 0.28 cm^2^. Response to 120 kVp X-rays at SSD = 100 cm: (**a**) Signal as a function of time for 100 mA and different voltages applied; (**b**) current–voltage curve under irradiation; (**c**) signal as a function of time for 25 mA and different mAs at 0.9 *V*/*V_W_*; (**d**) mA dependence for *V* = 0, 0.9 *V_W_* (2–20 mA (dashed line) delivered in fluoroscopic mode, 25–200 mA (solid lines) in radiography mode).

In Figure 6, the response of an FTF-TaC anodized at the formation voltage of *V_f_* = 100 V is presented. Figure 6a shows the signal as a function of time under irradiation with 120 kVp, 100 mA, 400 mAs, for different voltages applied. The corresponding IV-curve under irradiation is shown in Figure 6b. Here, the signal as a function of time for different mAs (irradiation time) for 120 kVp and 25 mA and fixed voltage *V* = 0.9 *V_W_* is reported. Figure 6d shows the mA dependence under irradiation with 120 kVp for *V* = 0 and *V* = 0.9 *V_W_*. The minimum sensitivity for 0 V is about 25 mA, while for *V* = 0.9 *V_W_* a measurable signal is observed down to 2 mA. The disruption in the linearity around 20 mA arises from the switching of the X-ray source from fluoroscopic mode (2–20 mA) to radiography mode (25–200 mA).

## 4. Discussion and Conclusions

Our results confirm prior findings of bulk photovoltaic effect in metal–insulator-metal junctions where the contact potential difference between metals is the driving mechanism [19,26] and pave the way for the development of large area self-powered radiation sensors. The use of Ta-Ta_2_O_5_ and other metal-metal-oxide electrodes as dosimeters was proposed by Feates and Knight in 1961 [20] but abandoned due to complexities of the measuring system and small signal compared to the background noise. Several prior works focused on the radiation hardness of Ta capacitors rather than on their customization as radiation sensitive devices [15,16,17,19,21].

We have demonstrated photovoltaic (null external voltage) and photoconductive (external voltage) X-ray induced currents in Ta_2_O_5_ layers for large area (>1 cm^2^) flat thin-film (TFT-TaC) and nano-porous sintered Ta (NP-TaC) capacitors. Considerable differences in NP-TaC vs. TFT-TaC geometries (and therefore kVp X-ray absorption) were accounted for, giving rise to similar temporal and IV-curve characteristics.

Photocurrents of up to about 50 nA were measured, with linear dependence on X-ray flux and on the total area of the Ta electrode for an NP-TaC active area of 16 cm^2^, and with linear-saturation-like dependence on the voltage bias. Temporal dependence revealed a characteristic RC increase of signal with a characteristic post-irradiation tail. For NP-TaC capacitors, the X-ray dose in the deeper layers is greatly diminished due to considerable attenuation of kVp X-rays in the shallow layers. This decrease in the magnitude of the signal with the depth is not present in the TFT-Ta.

We investigated the response under irradiation of several electrolyte tantalum capacitors. These devices are fabricated by anodization of tantalum pellets with a nano-porous structure and therefore they yield a very large total surface area. The capacitance and dark leakage current of these devices are therefore larger. The response under X-ray irradiation with a dose rate of ~2.9 cGy/s is relatively large (up to 50 nA for a 22 μF, 10 V capacitor). Tantalum absorbs 95% of 120 kVp radiation in about 350 μm, which is a small fraction of the pellet dimension, and the remaining volume absorbs rather than contributes to RIC. We, therefore, estimate that the signal under irradiation could be even larger for parallel plane TFT-Ta structures of the same total area. Of notice is the response of the capacitor to radiation at null external voltage (photoconductive region of IV-curve). This is due to two effects: (a) ionization of Ta_2_O_5_ by X-rays and charge carrier motion in the contact potential of about 0.5–1 V between the Ta and MnO_2_/polymer electrodes; (b) fast electrons escaping the Ta electrode or High Energy Current (HEC) [26,27,28,29].

The anodization of large flat thin-film tantalum foils resulting in FTF-TaC capacitors gives rise to self-powered thin film radiation sensors. Potential applications of such photosensitive structures are in medical, radiation safety, national security and space applications.

## Data Availability

The data presented in this study are available on request from the corresponding author.
